# Design and Computational Validation of γ-Ray Shielding Effectiveness in Heavy Metal/Rare Earth Oxide–Natural Rubber Composites

**DOI:** 10.3390/polym16152130

**Published:** 2024-07-26

**Authors:** Yongkang Liu, Xiaopeng Li, Yilin Yin, Zhen Li, Huisheng Yao, Zenghe Li, Heguo Li

**Affiliations:** 1 College of Chemistry, Beijing University of Chemical Technology, Beijing 100029, China; 2022210680@buct.edu.cn (Y.L.); yinyilin361@163.com (Y.Y.); 2023210201@buct.edu.cn (H.Y.); 2 State Key Laboratory of NBC Protection for Civilian, Beijing 100191, China; lxpbuct@163.com (X.L.); 18656433772@163.com (Z.L.)

**Keywords:** natural rubber, Geant4, WinXCom, radiation shielding properties, heavy metals oxide, rare earth oxide

## Abstract

This study involved the preparation of natural rubber-based composites incorporating varying proportions of heavy metals and rare earth oxides (Sm_2_O_3_, Ta_2_O_5_, and Bi_2_O_3_). The investigation analyzed several parameters of the samples, including mass attenuation coefficients (general, photoelectric absorption, and scattering), linear attenuation coefficients (μ), half-value layers (HVLs), tenth-value layers (TVLs), mean free paths (MFPs), and radiation protection efficiencies (RPEs), utilizing the Monte Carlo simulation software Geant4 and the WinXCom database across a gamma-ray energy spectrum of 40–150 keV. The study also compared the computational discrepancies among these measurements. Compared to rubber composites doped with single-component fillers, multi-component mixed shielding materials significantly mitigate the shielding deficiencies observed with single-component materials, thereby broadening the γ-ray energy spectrum for which the composites provide effective shielding. Subsequently, the simulation outcomes were juxtaposed with experimental data derived from a ^133^Ba (80 keV) γ-source. The findings reveal that the simulated results align closely with the experimental observations. When compared to the WinXCom database, the Geant4 software demonstrates superior accuracy in deriving radiation shielding parameters and notably enhances experimental efficiency.

## 1. Introduction

Being a potent source of renewable energy, nuclear power surpasses traditional fossil fuels in energy efficiency and lacks the greenhouse gas emissions associated with the latter, thereby having broad prospects for both military and civilian applications. However, alongside the benefits, nuclear energy also poses potential radiation risks, including exposure to γ- (gamma) and X-rays. To mitigate radiation’s threat to humans and other living organisms, researchers are developing innovative, cost-effective, lightweight, and non-toxic materials for radiation protection [1]. 

γ- (gamma) and X-ray radiation protection materials typically comprise organic and inorganic polymers, into which shielding fillers are integrated via physical mixing to fabricate composites with radiation shielding capabilities. Traditional γ- (gamma) and X-ray shielding equipment primarily utilizes lead-based materials, renowned for their superior shielding efficacy due to their high density and atomic number. However, lead’s biological toxicity and its detrimental environmental and biological impacts are increasingly being recognized. Consequently, materials that pose less risk to health and the environment are progressively replacing lead in radiation protection applications [2]. For instance, Varsha Agrawal et al. developed X-ray shielding tiles exhibiting robust impact resistance and a bending strength of 25 N/mm² by incorporating specific ratios of barium sulfate and a binder into red mud. These tiles present a viable alternative to traditional lead plates in X-ray diagnostics and CT imaging applications [3]. Akarapong Tuljittraporn et al. developed flexible X-ray shielding composites from thermoplastic natural rubber (TPNR), which is based on poly(butylene adipate) (PBAT) and natural rubber (NR), through the incorporation of antimony oxide [4]. Donruedee Toyen et al. developed multi-layer composites of natural rubber and Bi_2_O_3_, and, relative to the monolayer counterparts, the developed composites exhibited enhancements in elongation at break, tensile strength, and X-ray shielding capabilities to varying extents [5]. Abbas Bagheri Khatibani et al. synthesized samarium oxide/graphene nanostructures using the hydrothermal method; the results indicated that the composite material has a half-value layer of 0.16 cm, effectively protecting against gamma radiation [6]. Muddiyada Vishnu Muthamma et al. produced Ta_2_O_5_ and Ta_2_O_5_/Bi_2_O_3_ materials through die casting, which were then combined with epoxy resin to create gamma radiation shielding materials; the findings demonstrate that the addition of Ta_2_O_5_ significantly enhances the shielding performance of the materials compared to pure epoxy resin [7].

Currently, research into γ-ray shielding materials predominantly relies on experimental approaches. However, the nuclear radiation involved poses potential hazards to researchers, and the associated testing costs are considerable. Consequently, many researchers are turning to simulation software to aid in the design of radiation shielding materials. For instance, W. Chaiphaksa et al. developed natural rubber composites doped with SnO_2_ and utilized the WinXCom nuclear database, SRIM software (Version 2008), and the Phy-X program to calculate γ-ray shielding parameters, mass stopping power (MSP), the projected range (PR), and the fast neutron shielding removal cross section (FNSR) for protons and α-particles [8]. Basanta Subedi et al. conducted simulations and calculations to determine the attenuation coefficient (µ_m_/µ), the half-value layer and the tenth-value layer (HVL/TVL), the mean free path (λ), the effective atomic number (Zeff), the buildup factor (EBF/EABF), and the fast neutron removal cross section (Σ_R) for titanium-bearing metallic glass, utilizing Phy-X/PSD online platform and SRIM software [9]. L. Gerward et al. utilized the WinXCom software (Version 1.0.1) to model elemental substances and compounds, subsequently deriving and comparing the mass attenuation coefficients for γ- (gamma) and X-rays across various energy levels [10]. Jamila S. Alzahrani et al. investigated the γ-ray shielding properties of alloys based on Ni, Fe, Pb, and W across an energy range of 0.284 to 1.275 MeV using FLUKA software (Version 4.1) [11]. Ali H. Taqi et al. calculated and verified the γ-ray shielding properties of alloys based on Pb, Sn, and Cu using the Geant4 software (Version 11.1.1) and the XCOM database. The findings indicated that the Geant4-simulated data closely aligned with both the XCOM simulations and the experimental observations [12].

To develop gamma-ray shielding materials that offer enhanced protective effectiveness while optimizing cost-effectiveness, this study evaluated the γ-ray shielding efficiency of natural rubber composites doped with single- and multi-component fillers (Ta_2_O_5_, Bi_2_O_3_, and Sm_2_O_3_) across the 40–150 keV energy range. Analyses conducted using Geant4 software and the WinXCom database covered a range of parameters, such as mass and linear attenuation coefficients, half- and tenth-value layers, mean free paths, photoelectric absorption, and scatter-related metrics. The analysis, predicated on calculation results, elucidated discrepancies between the WinXCom database and the Geant4 software during the simulation process. Subsequently, experimental tests employing a ^133^Ba (80 keV) γ-ray source were conducted to validate the precision of the simulation data from Geant4 and calculations from the WinXCom database.

## 2. Design Theory of γ-Ray Shielding Materials

### 2.1. Principle of γ-Ray Shielding

**Figure 1:**
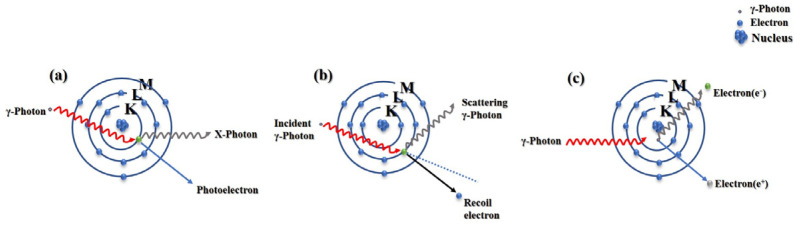
The mechanism of interaction between γ-photons and nuclei. (**a**) Photoelectric effect. (**b**) Compton scattering. (**c**) Electron-pair production.

Gamma rays are primarily attenuated and absorbed through the photoelectric effect with atoms, Compton scattering, and pair production [13]. The photoelectric effect manifests in two principal forms: ① γ-photons impart all their energy to extranuclear electrons, resulting in their emission from atoms as Auger electrons; ② γ-photons interact with atomic nuclei, placing atoms in an excited state. Subsequent transitions of outer electrons to internal vacancies are marked by the emission of characteristic X-rays. The probability of the photoelectric effect occurring is primarily influenced by the incident photon energy, the material’s density, and its atomic number [14]. Compton scattering is characterized by the inelastic interaction between photons of specific energy and loosely bound extranuclear electrons, which transfers a portion of the energy to electrons, leading to a decrease in photon energy, an increase in wavelength, and deflection from the incident direction at a specific angle. The electron-positron pair production effect commonly occurs during the interaction of high-energy gamma photons with atomic nuclei, under the influence of the nuclear electrostatic force, an incident gamma photon is converted into a pair of electron and positron. Ultimately, these pairs are annihilated, producing two photons of identical energy but moving in opposite directions. Figure 1 provides a schematic representation of the interaction between gamma rays and matter. Additionally, γ-rays can also undergo coherent scattering with atomic nuclei and be shielded. Generally, the energy of γ-rays is higher than the binding energy of electrons. Under the influence of the electric field of extranuclear electrons, the wavelength of γ-rays remains unchanged, and they are scattered out at a certain angle from the initial direction. Unlike the photoelectric effect and Compton scattering, low-energy gamma rays are more likely to undergo coherent scattering with high-atomic number elements, resulting in attenuation [15].

**Figure 2:**
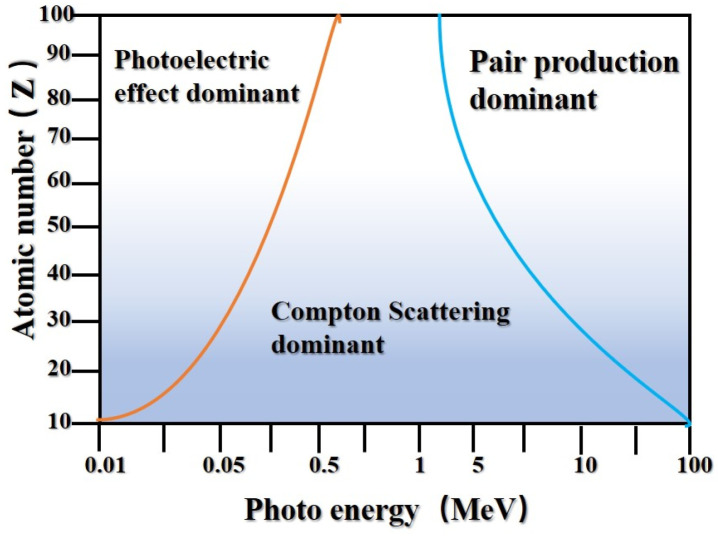
Variation of three types of interactions with γ-photon energy and atomic number.

Figure 2 illustrates the relationship between the γ-photon energy and the atomic number of metal atoms in influencing the occurrence of the photoelectric effect, Compton scattering, and pair production as mechanisms of gamma-ray interaction with matter. The three principal interaction mechanisms between γ-rays and atoms—the photoelectric effect, Compton scattering, and pair production—are each distinctly influenced by atomic number and incident photon energy. The photoelectric effect predominantly occurs during interactions between γ-photons and matter within the low energy range (<0.5 MeV). As photon energy increases (0.5–1 MeV), Compton scattering becomes the dominant interaction mechanism. In the high-energy realm (>1 MeV), γ-photons primarily undergo pair production, resulting in the creation of electron–positron pairs.

### 2.2. Theoretical Calculation of Radiation Shielding

The capability of a material to shield γ-photons can be assessed through several parameters: radiation protection efficiency (RPE), the linear attenuation coefficient (μ), the mass attenuation coefficient (μ_m_), the half-value layer (HVL), the tenth-value layer (TVL), the mean free path (MFP), and scattering attenuation efficiency (SAE); the discrepancy between simulation and experimental data is quantified as the deviation percentage (Dev%).

Radiation protection efficiency (RPE) represents the most direct measure of a sample’s radiation shielding capabilities [16]:

RPE = (N_0_ − N)/N_0_ × 100%

The attenuation coefficient serves as a crucial metric for assessing the radiation shielding capacity of materials, primarily determined through Lambert–Beer’s law [17]:

Linear attenuation coefficient: μ = (InN_0_ − InN)/d;

Mass attenuation coefficient: μm = μ/ρ;

where N_0_ represents the photon energy detected without the presence of any shielding material, N denotes the photon energy detected after penetrating the shielding material, d represents the sample’s effective thickness, and ρ signifies the shielding material’s effective density.

Half-value layer: HVL = In2/μ;

Tenth-value layer: PVL = In10/μ;

The half-value layer (HVL) represents the thickness required for a material to reduce the radiation intensity to half of its initial value.

The tenth-value layer (TVL) is defined as the material thickness needed to attenuate the radiation intensity to one-tenth of its original level.

The mean free path (MFP) refers to the average distance a photon travels between two successive interactions [18]:

MFP = 1/μ

where μ represents the linear attenuation coefficient.

The discrepancy between Geant4 simulations and XCOM calculations is determined through the following equation [12]:

$$Dev\%=\frac{{\left(u_{m}\right)}_{XCOM}-{\left(u_{m}\right)}_{Geant4}}{{\left(u_{m}\right)}_{Geant4}}\times 100\%$$

The scattering probability (SSE) during gamma-photon attenuation in materials is determined by the following equations:

SSE = u_m(scattering)_/u_m_ ×100%

where u_m(scattering)_ = N_(scattering)_/(N_0_ − N) × u_m_ and N_(scattering)_ denotes the energy associated with scattering processes in radiation shielding.

The aforementioned equation will be utilized for the subsequent calculation of radiation shielding parameters of the material.

### 2.3. Analysis of Radiation Simulation Software

Given the extensive research conducted by many scholars, we have explored current nuclear radiation simulation software packages and highlighted the distinctions among them. Among these, the XCOM nuclear database, developed by the American National Standards Laboratory, stands out as the most frequently utilized program. To enhance user convenience, Gerward et al. transformed the web-based XCom database into a Windows-compatible PC program, subsequently rebranding it as WinXCom [19]. Users can configure interactions among diverse elements, compounds, mixtures, and γ-photons of varying energies, thereby determining the total mass attenuation coefficients for different combinations of elements relative to γ-photons. Commonly utilized nuclear radiation simulation programs include those based on the Monte Carlo method, as shown in Figure S1. Via extensive data sampling and analysis of particle–material interactions, including the quantification of subsequent energy and state alterations, vital information such as energy deposition and particle dose distribution within the material is ascertainable. Further, through analytical computation, the shielding properties of the barrier can be determined.

Table 1 summarizes the distinctive features and variances among the various simulation software packages evaluated. Geant4, developed in the C++ programming language, simulates the transport behavior of particles within matter by modeling the physical processes governing the interactions between particles and materials. Compared to other nuclear radiation simulation programs, Geant4 offers significant advantages in modeling high-energy particle interactions with matter, constructing geometric models, and facilitating 3D visualization. The WinXCom database offers mass attenuation coefficients for a wide array of substances and their combinations, facilitating the assessment of calculation accuracy and serving as a foundation for subsequent experimental endeavors. Based on the analysis and comparison of the various simulation software packages discussed earlier, we chose Geant4 and WinXCom to calculate the shielding performance of the materials.

## 3. Preparation of Materials and Methodologies for Simulating and Testing Shielding Performance

### 3.1. Preparation of Materials

In this study, bismuth oxide (Bi_2_O_3_), samarium oxide (Sm_2_O_3_), and tantalum pentoxide (Ta_2_O_5_) were chosen as γ-ray shielding components, with natural rubber serving as the polymer matrix. Rubber-based shielding composites were synthesized by incorporating either single or multiple metal oxide fillers into the mixture. The fabrication process was segmented into three distinct phases: internal mixing, open milling, and vulcanization pressing. Initially, 100 g of natural rubber was processed into a viscoelastic compound using an internal mixer. Subsequently, a blend containing 7 g of zinc oxide and stearic acid was incorporated. After thorough mixing, 40 g of carbon black and 1 g of antioxidant 264 were added to yield a mixed rubber devoid of any shielding filler. Subsequently, the prepared rubber mixture was transferred to an open mill. Sequentially, 200 g of the shielding filler was added, and thorough incorporation was ensured before introducing 1.6 g of an accelerator blend (comprising D, M, CZ, and DM). Following uniform blending, 1.5 g of sulfur was incorporated to yield a mixed rubber devoid of any shielding filler. Subsequently, the prepared rubber mixture was transferred to the open mill. Sequentially, 200 g of the shielding filler was added, and thorough incorporation was ensured before introducing 1.6 g of an accelerator blend (comprising D, M, CZ, and DM). Following uniform blending, 1.5 g of sulfur was incorporated to achieve a homogeneous mixture, resulting in the formation of an unvulcanized composite film. The prepared mixed film was placed into a mold measuring 13 mm in length and 11 mm in width. The curing compound was then produced using a plate curing press set to a temperature of 143 °C, a pressure of 18 MPa, and a curing duration of 8 min.

### 3.2. Design of the Simulation Experiment

**Figure 3:**
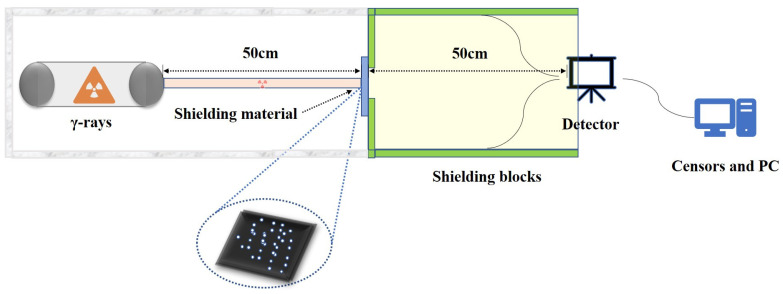
Schematic diagram of γ-ray shielding performance testing device.

Figure 3 illustrates the testing apparatus employed to evaluate the shielding efficacy against γ-rays in this study. The gamma-radiation absorption dose rate of the composite material was measured using a portable lanthanum bromide gamma spectrometer, and the radiation source used in the experiment was ^133^Ba (80 keV). The experimental setup was constructed according to the ISO 4037-1:2019 standard [24]. A filtering device confines the radioactive source particles within the specified measuring range. The detector first measures the baseline energy of gamma particles without any shielding material, denoted as N_0_. It then calculates the energy of gamma particles that penetrate the shielding material, denoted as N, and the difference between these measurements, N_0_ − N, represents the total energy effectively shielded by the material. The ratio of the total energy effectively shielded to the total energy of gamma particles without shielding material is defined as the shielding efficiency of the material against gamma radiation [25]. As illustrated in the schematic of the experimental setup in Figure 3, Figure S2 presents a Geant4-constructed 3D model of a shielding test. This model features a cuboidal shielding material with dimensions of 10 cm in both length and width, while its thickness is determined through experimental measurements. The distance from the radiation source to the shielding material is 50 cm, with a similar 50 cm gap between the shielding material and the detector. The experiment simulates 100,000 radiation particles, deploying γ-ray energies of 40 keV, 60 keV, 80 keV, 100 keV, and 150 keV. All components—the material, the detector, and the source—are enclosed within an airtight geometry composed of a lead plate, designed specifically for capturing γ-rays (X-rays) generated in experiments. The construction of shielding materials and their geometrical configuration is primarily facilitated by the DetectorConstruction and G4Material classes. Figure S3 illustrates the methodology for defining the geometry and materials utilized in fabricating a natural rubber composite doped with tantalum pentoxide composite shield.

## 4. Results and Discussion

To address the limited absorption efficiencies of various metals in radiation shielding and to broaden the energy range of materials for effective shielding, we selected three metals—samarium, tantalum, and bismuth—each with distinct K-edge absorption properties. Figure S4a illustrates the utilization of WinXCom’s built-in photon absorption cross-section data for heavy metals and rare earth elements, sourced from the elemental database. The K-edge absorption values for samarium, tantalum, and bismuth are, respectively, 46.83 keV, 67.42 keV, and 90.53 keV. Bismuth, with a K-edge absorption value close to that of lead, effectively shields against high-energy gamma rays; however, it exhibits a diminished absorption capability within the 40–80 keV energy range for gamma photons. Therefore, we chose tantalum and samarium elements to compensate for the shielding gaps of bismuth against low-to-medium-energy gamma photons. Additionally, due to the high cost of pure Bi, Ta, and Sm, their oxides (Bi_2_O_3_, Ta_2_O_5_, and Sm_2_O_3_) were used as gamma shielding components [24]. Shielding fillers were integrated into the natural rubber matrix via mechanical blending to enhance the composite’s overall γ-ray shielding efficacy. Figure S4b depicts the experimentally prepared natural rubber composite material doped with heavy metal/rare earth oxides. Table 2 presents the physical characteristics of the composite material as determined experimentally.

### 4.1. Comparative Analysis of Shielding Performance of Composite Materials Doped with Single and Multiple Shielding Fillers

**Figure 4:**
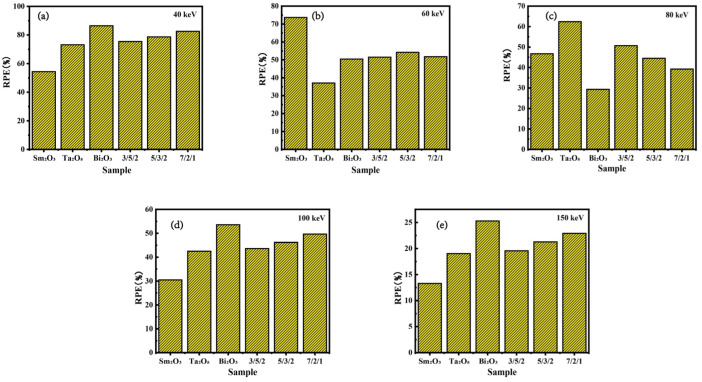
Generated through Geant4 simulations, these results delineate the radiation shielding efficiencies of composite materials composed of varying formulations at energies of (**a**) 40 keV, (**b**) 60 keV, (**c**) 80 keV, (**d**) 100 keV, and (**e**) 150 keV.

Figure 4a–e illustrate the effects of doping composite materials with single and multiple components (Sm_2_O_3_, Ta_2_O_5_, and Bi_2_O_3_) on their γ-ray shielding capabilities at various energies (40 keV, 60 keV, 80 keV, 100 keV, and 150 keV) as determined through simulations performed with Geant4. At gamma-ray energies of 40 keV, 100 keV, and 150 keV, composites incorporating multiple dopants demonstrate superior shielding effectiveness compared to composites doped exclusively with Sm_2_O_3_ or Ta_2_O_5_. Notably, at 60 keV, composites with multi-component doping show enhanced shielding performance compared to those doped solely with Ta_2_O_5_ or Bi_2_O_3_. Furthermore, at 80 keV, composites doped with a multi-component mixture exhibit significantly enhanced shielding capabilities in comparison to those exclusively doped with Sm_2_O_3_. The results indicate that multi-component composite shielding materials significantly compensate for the gamma-radiation shielding deficiencies observed in single-component materials, thereby extending the energy spectrum over which the composite materials provide effective gamma-radiation protection. However, within the energy ranges characterized by strong absorption peaks of certain elements, their shielding effectiveness is marginally inferior to that of single-component materials with equivalent compositional concentrations.

### 4.2. Simulation and Verification of Radiation Shielding Performance

**Figure 5:**
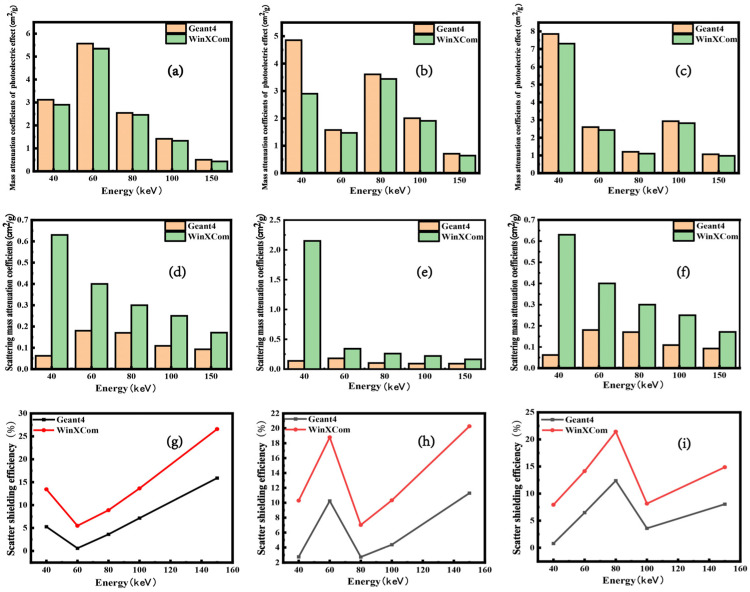
Mass attenuation coefficients due to photoelectric absorption (**a**–**c**), mass attenuation coefficients from scattering (**d**–**f**), and scattering efficiencies (**g**–**i**) of composites doped with samarium oxide, tantalum pentoxide, and bismuth oxide, as computed using Geant4 and WinXCom.

In the shielding of medium- and low-energy γ-photons, metal atoms predominantly engage in photoelectric absorption, with scattering being attenuated or absorbed, resulting in the generation of Auger electrons. Consequently, the total mass attenuation coefficient primarily consists of contributions from photoelectric absorption and scattering. Figure 5a–c illustrate the variation in mass attenuation coefficients due to photoelectric absorption across energy levels in composites doped with a single shielding filler as determined by Geant4 and WinXCom software simulations. The attenuation coefficients derived from the Geant4 simulations and the WinXCom database exhibit variations with incident photon energy; the overarching trend is consistent across both methods. Notably, when the incident γ-ray photon energy approaches the K-edge of the metal atoms, photoelectric absorption becomes the predominant mechanism [26]. At gamma-ray energies of 60 keV, 80 keV, and 100 keV, the mass attenuation coefficients for gel formulations doped with samarium oxide, tantalum pentoxide, and bismuth oxide exhibit significant increases, with photoelectric absorption predominating. Figure 5d–f illustrate the variation in scattering mass attenuation coefficients for composites with a single type of shielding filler, derived from simulations using Geant4 and WinXCom software. The Geant4 simulation outcomes reveal that the scattering mass attenuation coefficient initially rises with an increase in incident energy, followed by a subsequent decrease. Conversely, the WinXCom data demonstrate a consistent decrease in the scattering mass attenuation coefficient as incident energy increases, with the values continuously surpassing those obtained from Geant4 simulations. Figure 5g–i display the variation in scattering attenuation rates for gel formulations infused with samarium oxide, tantalum pentoxide, and bismuth oxide, plotted against gamma-photon energy as determined by Geant4 and WinXCom simulations. The findings reveal a basic agreement in the trend of scattering shielding effectiveness with respect to gamma-photon energy between the Geant4 and WinXCom simulations, with the simulated values from Geant4 being marginally lower than those from WinXCom.

**Figure 6:**
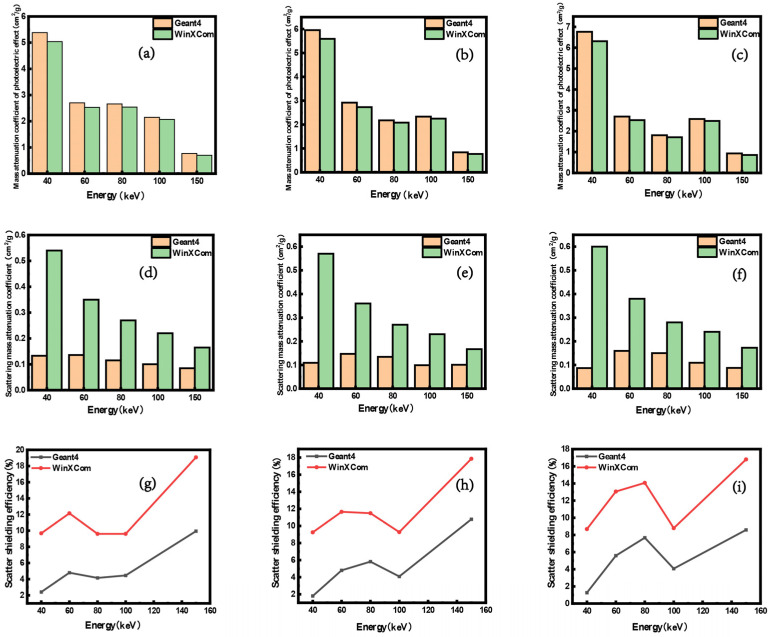
Mass attenuation coefficients due to photoelectric absorption (**a**–**c**), mass attenuation coefficients from scattering (**d**–**f**), and scattering efficiencies (**g**–**i**) of composites with mixed components in ratios of 3/5/2, 5/3/2, and 7/2/1, as computed using Geant4 and WinXCom.

Figure 6a–c depict the energy-dependent variations in mass attenuation coefficients attributed to photoelectric absorption in the composite materials. The trends of the values computed by Geant4 and WinXCom closely align with the changes in energy. Additionally, with increasing incident gamma-photon energy, the differences between the two datasets gradually converge. Figure 6d–f depict the variation in mass attenuation coefficients due to scattering within composite materials as a function of energy. The simulations conducted via Geant4 indicate that the scattering mass attenuation coefficients initially increase with rising energy levels, only to decrease thereafter. Conversely, within WinXCom, these coefficients consistently diminish as energy escalates. Notably, the discrepancy between the two computational approaches lessens with the increment in gamma energy. In the Geant4 simulations, when low-energy gamma rays interact with metal atoms, the photoelectric effect’s contribution surpasses that predicted by the WinXCom database, while the scattering contributions fall short of WinXCom’s simulations. Figure 6g–i present the curves depicting the variations in scattering attenuation rates of gel composites, formulated at different ratios (3/5/2, 5/3/2, and 7/2/1), against gamma-photon energy, as independently predicted by the Geant4 and WinXCom simulations. It is noteworthy that the gamma-photon energy-dependent trends for the composites, which incorporate various doping agents, demonstrate significant congruity as analyzed by both Geant4 and WinXCom. The findings reveal that, within composites doped with either single or multiple fillers, the WinXCom software predicts higher probabilities of scattering during the gamma-ray shielding process compared to Geant4, whereas it anticipates lower probabilities of photoelectric absorption.

**Figure 7:**
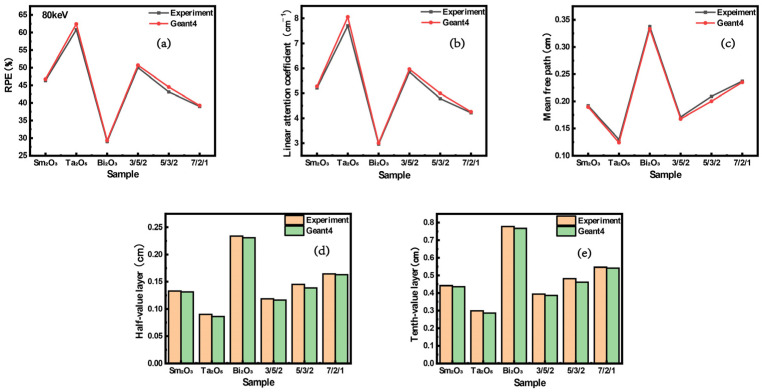
The shielding performance of materials with different compositions as determined through Geant4 simulations and experimental testing. (**a**) Radiation protection efficiencies. (**b**) Linear attenuation coefficients. (**c**) Mean free path values. (**d**) Half-value layers. (**e**) Tenth-value layers.

Figure 7a presents the shielding efficiency metrics for materials comprising varied constituents. The divergence between the experimental and calculated values remains within 1.63%. Considering that the 80 keV energy spectrum aligns with bismuth’s absorption blind spot [27], composite materials doped with bismuth oxide exhibit suboptimal shielding properties. However, the incorporation of multiple components into the composite material enhanced its shielding capabilities to some extent. Figure 7b–e respectively illustrate the linear attenuation coefficients, mean free path values, half-value layers, and tenth-value layers for materials of varying compositions. The results indicate that composite materials doped with tantalum pentoxide exhibit the best shielding performance, with values calculated by Geant4 aligning closely with experimental test data.

Table 3 presents the mass attenuation coefficients obtained under a ^133^Ba gamma radiation source at 80 keV as determined through experimental testing, Geant4 computation, and WinXCom simulation. At 80 keV, the calculations by Geant4, data from the WinXCom database, and experimental measurements show substantial concordance. Specifically, the discrepancy between Geant4’s calculations and the experimental results does not exceed 4.920%, while the difference between WinXCom’s data and the experimental findings is no more than 6.220%. As indicated in the table, the numerical data derived from the Geant4 simulations are closer to the experimental results compared to those obtained from WinXCom, with the simulated values being slightly higher than the experimental outcomes. This discrepancy is primarily due to the WinXCom simulation. At 80 keV, calculations by Geant4, data from the WinXCom database, and experimental measurements show substantial concordance. In this simulation, we ignored trace processing aids (such as accelerators, anti-aging agents, and stearic acid) that have negligible effects on the shielding performance of the composite materials. Consequently, the calculated shielding performance of the composite material was slightly overestimated compared to the experimental values.

## 5. Conclusions

This study employs the Geant4 simulation program and the WinXCom database to perform computational analyses on natural rubber doped with single-component Sm_2_O_3_, Ta_2_O_5_, and Bi_2_O_3_, as well as multi-component mixtures, across gamma-ray energies ranging from 40 to 150 keV. The simulation outcomes were compared against experimental data obtained from a ^133^Ba (80 keV) gamma-radiation source, demonstrating fundamental agreement between the simulated results and the empirical findings. Multi-component composite shielding materials substantially mitigate the gamma-radiation shielding blind spots inherent to single-component materials, thereby broadening the energy range across which composite materials effectively shield against gamma radiation. However, within the energy bands of strong absorption by specific elements, their shielding efficacy is lower compared to that of single-component materials with equivalent constituent concentrations. At lower energy levels, the Geant4 computational outcomes reveal that the proportion of the photoelectric effect occurring during particle interaction with materials surpasses the simulated values derived from the WinXCom database, whereas the scattering proportion falls below the simulation results of WinXCom. However, as the energy of gamma photons increases, the discrepancy between the two gradually diminishes. Under gamma radiation at an energy level of 80 keV, data calculated using Geant4 align more closely with experimental results compared to values obtained from WinXCom. This indicates that, under identical conditions, Geant4 offers superior accuracy in its computational outcomes relative to WinXCom simulations, effectively calculating the radiation shielding parameters of materials.

## Figures and Tables

**Table 1:** Characteristics of and differences between Monte Carlo simulation software packages.

	Advantages	Shortcomings
Geant4-11.1.1 [20]	Boasts a robust 3D visual interface, facilitating a broad spectrum of applications and the capacity to navigate complex physical processes	Presents a steep learning curve for beginners, necessitating extended learning periods and advanced computer hardware specifications
SuperMC-3.3.0 [21]	Demonstrates marked practicability and computational efficiency in specialized nuclear radiation domains	Its applicability in certain domains is constrained, and it lacks sufficient international recognition compared to other software
FLUKA-4.1 [22]	Capable of simulating a diverse array of particle interaction processes	Users are required to possess knowledge in physics and computing, with the graphical interface leaving room for improvement
EGS-4 [23]	Offers exceptionally high simulation accuracy for electrons and photons	Its simulation capabilities for other particle types are less robust
MCNP-5 [22]	This tool is extensively adopted in radiation protection, offering practical neutron simulation capabilities	Encounters limitations when addressing intricate physical phenomena

**Table 2:** Physical parameters of rubber matrix composites with varying components.

Sample	Filling Composition (200 g)	Density (g/cm^3^)	Thickness (cm)
1	Sm_2_O_3_	1.997	0.1194
2	Ta_2_O_5_	2.172	0.1215
3	Bi_2_O_3_	2.182	0.1157
4	Bi_2_O_3_/Ta_2_O_5_/Sm_2_O_3_(3/5/2)	2.149	0.1186
5	Bi_2_O_3_/Ta_2_O_5_/Sm_2_O_3_(5/3/2)	2.162	0.1179
6	Bi_2_O_3_/Ta_2_O_5_/Sm_2_O_3_(7/2/1)	2.175	0.1172

**Table 3:** Mass attenuation coefficients of composite materials with varied compositions as determined by Geant4, WinXCom, and experimental measurements.

γ-Ray Energy (80 keV) Mass Attenuation Coefficients (cm^2^/g)					
Sample	Experimental Value	Geant4	Dev (%)	WinXCom	Dev (%)
Sm_2_O_3_	2.584 ± 0.022	2.643	2.28 ± 0.86	2.700	4.49 ± 0.88
Ta_2_O_5_	3.593 ± 0.031	3.709	3.23 ± 0.86	3.700	2.98 ± 0.86
Bi_2_O_3_	1.333 ± 0.017	1.376	3.23 ± 1.30	1.400	5.03 ± 1.33
3/5/2	2.673 ± 0.028	2.776	3.85 ± 1.07	2.810	5.13 ± 1.09
5/3/2	2.283 ± 0.010	2.311	1.23 ± 0.44	2.350	2.94 ± 0.45
7/2/1	1.918 ± 0.013	1.955	1.93 ± 0.69	1.990	3.75 ± 0.69

## Data Availability

The original contributions presented in the study are included in the article/Supplementary Material, further inquiries can be directed to the corresponding authors.

## Supplementary Materials

The following supporting information can be downloaded at: https://www.mdpi.com/article/10.3390/polym16152130/s1, Figure S1: Commonly used radiation simulation software; Figure S2: 3D shielding test model built by Geant4; Figure S3: Method of defining shield geometry and material in Geant4; Figure S4: (a) K-Layer absorption edge values of shielding elements in composite materials. (b) Experimentally prepared composite rubber.
