# Supplementary material for: Design and Computational Validation of γ-Ray Shielding Effectiveness in Heavy Metal/Rare Earth Oxide–Natural Rubber Composites
Source: Polymers (Basel). 2024 Jul 26;16(15):2130. doi: 10.3390/polym16152130 (PMC11314579; doi:10.3390/polym16152130)
Supplement: Supplementary file 1 [file polymers-16-02130-s001.zip › polymers-3035280-supplementary.pdf]

polymers

MDPI

Article

Supplemental Information for

Design and Computational Validation of γ-Ray Shielding Effectiveness in Heavy Metal/Rare Earth Oxide-Natural Rubber Composites

Yongkang Liu [1], Xiaopeng Li [2], Yilin Yin [1], Zhen Li [2], Huisheng Yao [1], Zenghe Li [1,*] and Heguo Li [2,*]

[1] College of Chemistry, Beijing University of Chemical Technology, Beijing 100029, China; 2022210680@buct.edu.cn (Y.L.); yinyilin361@163.com (Y.Y.); 2023210201@buct.edu.cn (H.Y.)
[2] State Key Laboratory of NBC Protection for Civilian, Beijing 100191, China; lxpbuct@163.com (X.L.); 18656433772@163.com (Z.L.)
* Correspondence: lizh@mail.buct.edu.cn (Z.L.); liheguo1972@126.com (H.L.); Tel.: +86-010-64435714 (Z.L.)

Citation: Liu, Y.; Li, X.; Yin, Y.; Li, Z.; Yao, H.; Li, Z.; Li, H. Design and Computational Validation of γ-Ray Shielding Effectiveness in Heavy Metal/Rare Earth Oxide–Natural Rubber Composites. *Polymers* **2024**, *16*, 2130. https://doi.org/10.3390/polym16152130

Academic Editor: Alexey V. Lyulin

Received: 15 May 2024
Revised: 9 July 2024
Accepted: 17 July 2024
Published: 26 July 2024

Copyright: © 2024 by the authors. Submitted for possible open access publication under the terms and conditions of the Creative Commons Attribution (CC BY) license (https://creativecommons.org/licenses/by/4.0/).

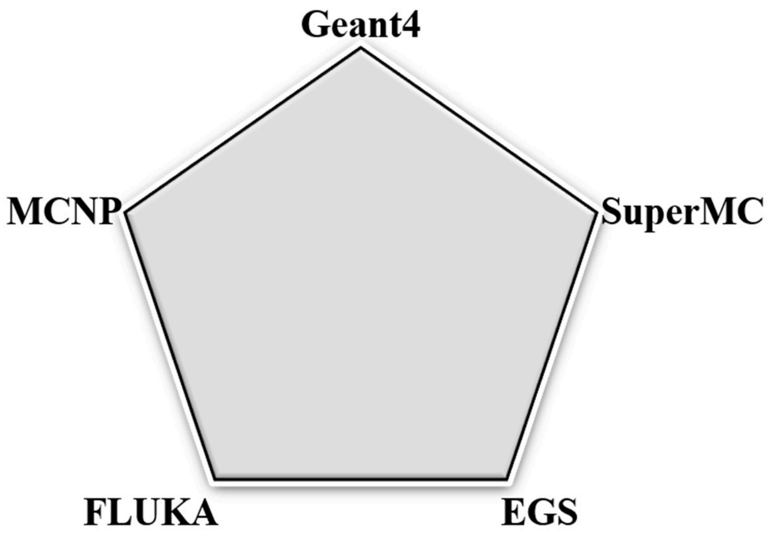

Figure S1. Commonly used radiation simulation software

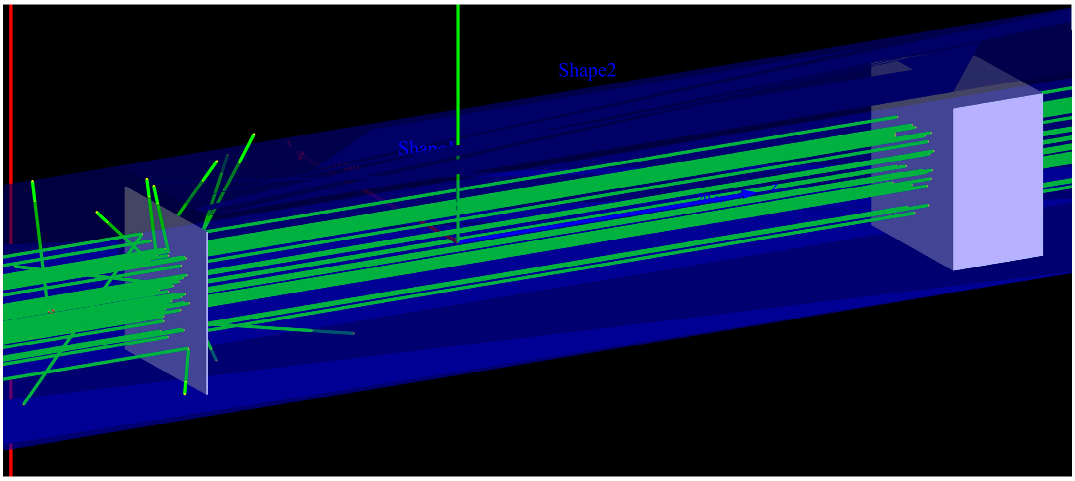

Figure S2. 3D shielding test model built by Geant4

```
G4ThreeVector pos1 = G4ThreeVector(0, 0,-4.8*cm);
G4double material_dxa=10*cm, material_dxb=10*cm;
G4double material_dya=10*cm, material_dyb=10*cm;
G4double material_dz=10*cm;
G4Material*C5H8CTa2O5ZnO=new G4Material("CHCTaZn", 2.172*g/cm3,ncomponents=5);
C5H8CTa2O5ZnO ->AddElement(C,fractionmass=37.2*perCent);
C5H8CTa2O5ZnO ->AddElement(O,fractionmass=10.8*perCent);
C5H8CTa2O5ZnO ->AddElement(H,fractionmass=3.40*perCent);
C5H8CTa2O5ZnO ->AddElement(Zn,fractionmass=1.10*perCent);
C5H8CTa2O5ZnO ->AddElement(Ta,fractionmass=47.5*perCent);
```

Figure S3. Method of defining shield geometry and material in Geant4

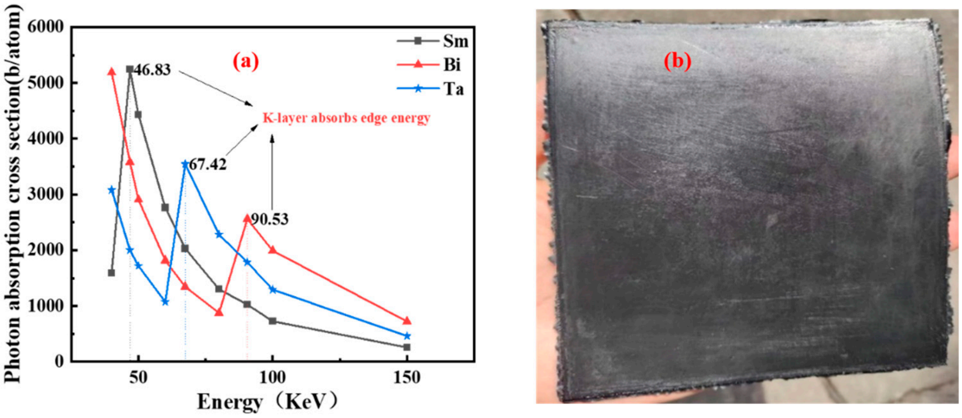

Figure S4. (a) K-Layer absorption edge values of shielding elements in composite materials (b) Experimentally prepared composite rubber
